# Secure and Privacy Enhanced Gait Authentication on Smart Phone

**DOI:** 10.1155/2014/438254

**Published:** 2014-05-14

**Authors:** Thang Hoang, Deokjai Choi

**Affiliations:** Department of Electronics and Computer Engineering, Chonnam National University, Gwangju 500-757, Republic of Korea

## Abstract

Smart environments established by the development of mobile technology have brought vast benefits to human being. However, authentication mechanisms on portable smart devices, particularly conventional biometric based approaches, still remain security and privacy concerns. These traditional systems are mostly based on pattern recognition and machine learning algorithms, wherein original biometric templates or extracted features are stored under unconcealed form for performing matching with a new biometric sample in the authentication phase. In this paper, we propose a novel gait based authentication using biometric cryptosystem to enhance the system security and user privacy on the smart phone. Extracted gait features are merely used to biometrically encrypt a cryptographic key which is acted as the authentication factor. Gait signals are acquired by using an inertial sensor named accelerometer in the mobile device and error correcting codes are adopted to deal with the natural variation of gait measurements. We evaluate our proposed system on a dataset consisting of gait samples of 34 volunteers. We achieved the lowest false acceptance rate (FAR) and false rejection rate (FRR) of 3.92% and 11.76%, respectively, in terms of key length of 50 bits.

## 1. Introduction

Smart environments established by the development of mobile technology have brought vast benefits to human being [1]. Nowadays, mobile devices could be utilized not only for communication and entertainment but also for transaction [2], personal healthcare [3], or even in emergency situations [4]. As a result, more and more personal data are collected and kept in the mobile device for analysis [5], which would lead to increasing system security and user privacy concerns. Basically, security techniques for authentication and identification are commonly based on password (e.g., OTP [2]), token (e.g., ID cards), or biometric recognition (e.g., iris [6], fingerprint [7], face [8], and gait [9] recognition). Biometric based authentication mechanisms are more convenient in terms of end-user usage viewpoint when comparing with the two remaining methods of password and token. However, using biometric authentication on mobile devices should be considered carefully. Due to the fact that biometrics is unique but fuzzy and revocable, most conventional biometric authentication systems are developed based on pattern recognition and machine learning (PR-ML) algorithms to deal with the natural variations of biometric measurement [6]. Enrollment biometric templates or extracted features are stored under unconcealed form for matching with a new biometric sample to authenticate/identify users. This kind of approaches could leave critical vulnerabilities in terms of system security and user privacy, especially when it is implemented on mobile devices. These devices are easily lost so that an adversary could illegally access the mobile repository to obtain original biometric templates. Since biometrics is tied to unique characteristics of an individual which are hardly changed, the user privacy leak means an adversary could partly or fully determine the user's biometrics. From the viewpoint of system security, a compromise of biometric templates results in everlasting forfeiture. An adversary could utilize compromised templates to thereafter always illegally grant access to sensitive services.

In this paper, we introduce an authentication system based on biometric cryptosystem (BCS) to enhance the system security and user privacy on mobile devices. The biometric modality used in our system is human gait which is collected using an inertial sensor named accelerometer attached to the user's body. This type of sensor has been utilized to propose motivating applications in smart phones recently [3]. To the best of our knowledge, this is the first approach of a BCS using gait biometrics captured from the accelerometer. We utilize a fuzzy commitment scheme [10] whereby the key, acting as an authentication factor, is biometrically encrypted by the user's gait. The gait sample is merely employed to retrieve the cryptographic key and then be always discarded so that the system security and user privacy are significantly enhanced. Moreover, the system has significant advantages in terms of small storage space and low computational requirements. Therefore, it is more applicable to be deployed directly on mobile devices with limited resources, compared with other PR-ML based systems [9].

The rest of this paper is organized as follows.  Section 2 presents the related works. Our proposed system is described in Section 3. Experimental evaluations are presented in Section 4. Finally, Section 5 draws our conclusions.

## 2. Related Works

To preserve the security and user privacy of biometric authentication systems, various modern approaches have been proposed [11], wherein biometric cryptosystems (BCSs) have attracted much research in recent years. State-of-the-art BCSs which were previously proposed mostly utilize physiological modalities such as iris [12], face [13], and fingerprint [14]. There are some studies that use behavioral biometrics such as signature [15] and voice [16]. Generally, BCSs could be classified into 2 subsystems including key-binding and key-generation systems [11]. In key-binding systems, a random key string is generated and then bound with a biometric template yielding helper data. Such data are stored for further utilization to retrieve the key in the authentication phase. For example, Hao et al. [17] proposed an iris based BCS using fuzzy commitment scheme. They used 2048 bits of iris code combined with concatenated codes and achieved the false acceptance rate (FAR) and false rejection rate (FRR) of 0% and 0.47%, respectively, and the key length of their system is 140 bits. In contrast to key-binding systems—the key generation scheme—helper data is created directly only from the biometric template. Such helper data will associate with a presented query which is sufficiently close to the original template to generate either the unique key string or the original template. Typical techniques of such scheme are fuzzy extractor [18] and secure sketches [19]. Applications of key-generated scheme have already been implemented on iris [12] and voice [16]. Generally, approaches on physiological modalities achieved better results in terms of error rates and security level, compared with behavioral biometric factors. This is due to the fact that physiological modalities such as iris and fingerprint are more robust than behavioral factors which are significantly affected by various conditions. For example, human voice depends on the state of health, gait of individual changes over time, and so forth.

## 3. The Proposed Method

**Figure 1:**
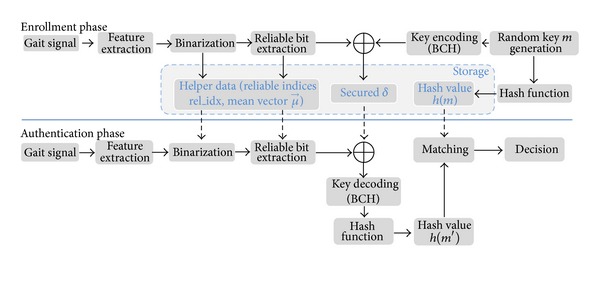
The overall architecture of our proposed gait based BCS using a fuzzy commitment scheme where *⊕* denotes the exclusive-OR operator.

Figure 1 sketches the specification of our gait based BCS using a fuzzy commitment scheme [10]. In the enrollment phase, gait signal of a user *U* will be acquired and preprocessed to reduce the influence of the acquisition environment. Feature vectors are extracted in both time and frequency domains and then binarized. After that, a reliable binary feature vector *ω* is extracted based on determining reliable components. Concurrently, a cryptographic key *m*, which is generated randomly corresponding to each user, is encoded to a codeword *c* by using error correcting codes. The fuzzy commitment scheme *F* computes the hash value of *m* and a secured *δ* using a cryptographic hash algorithm *h* and a binding function, respectively. The helper data which are used to extract reliable binary feature vectors and values of *h*(*m*), *δ* are locally stored for later use in the authentication phase.

In the authentication phase, the user supposed to be *U* will provide a different gait sample. It is also preprocessed to extract a feature vector and a reliable vector *ω'* is extracted by using helper data which is previously stored in the enrollment phase. The decoding function *f* computes the corrupted codeword *c*′ via binding *ω*′ with *δ* and then retrieves a cryptographic key *m*′ from *c*′ using a corresponding error correcting code decoding algorithm. Finally, the hash value of *m'* will be matched with *h*(*m*) for authentication decision.

### 3.1. Gait Signal Preprocessing and Segmentation

#### 3.1.1. Data Acquisition

**Figure 2:**
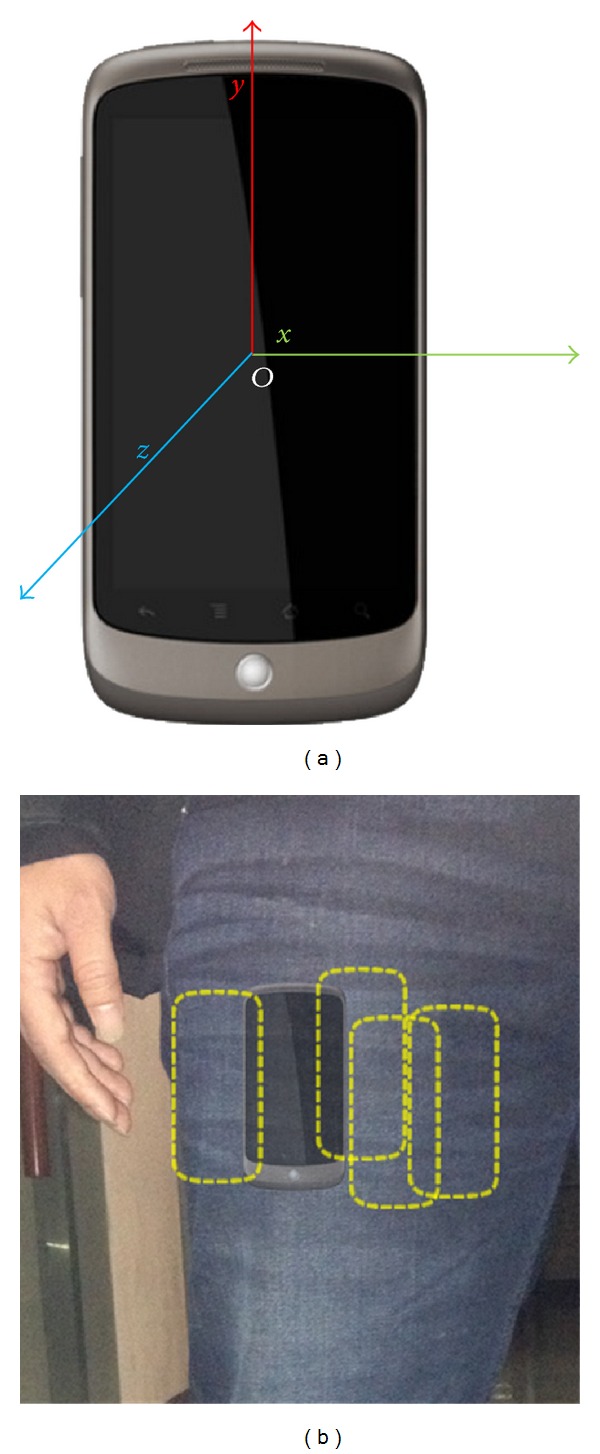
(a) Google Nexus One phone with a built-in 3-axial accelerometer and (b) the position of device put inside the front trouser pocket.

A Google Nexus One smart phone put inside front pocket is employed to collect user gait signal (Figure 2). This discrete time signal is a sequence of combined values of gravity acceleration, ground reaction force, and inertial acceleration which are captured by a built-in 3-dimensional accelerometer during walking. We present the output of this accelerometer as 3-component vectors

(1) $$\begin{array}{c} A=\left[a_{X},a_{Y},a_{Z}\right], \end{array}$$

where *a*_*X*_,  *a*_*Y*_,  *a*_*Z*_ represent the magnitude of the acceleration values acting on three directions, respectively.

#### 3.1.2. Data Interpolation

As the accelerometer integrated in mobile devices is power saving and designed to be simpler than standalone sensors, its sampling rate is not stable and entirely depends on mobile OS. The time interval between two consecutive returned samples is not a constant. The sensor only outputs value when the acceleration on 3 dimensions has a significant change. The sampling rate of Google Nexus One used in our study is instable and fluctuates around 27 ± 2 Hz. Therefore, acquired signal is interpolated to 32 Hz using linear interpolation to ensure that the time interval between two sample points will be fixed.

#### 3.1.3. Noise Filtering

When accelerometer samples movement data by user walking, some noises will inevitably be collected. These could be yielded by idle orientation shifts or bumps on the road during walking. Moreover, mobile accelerometer produces numerous noises compared with standalone sensors since its functionality is fully governed by mobile OS layer. Hence, we adopt a multilevel wavelet decomposition and reconstruction method, specifically the Daubechies orthogonal wavelet (*Db6*) with level 2, to filter the gait signal. In 1st level, original gait signal is decomposed into two separate parts containing coarse and detail coefficients. Such coarse coefficients acquired in the 1st level are then used as input signal to be decomposed in the next level. This process continues until the desired level is achieved. To eliminate the impacts of noise, in each level, we assign detail coefficients which are lower than a predefined threshold to 0. The noise-filtered signal is reconstructed conversely to the decomposition process, wherein coarse coefficients will associate with new detail coefficients starting from the lowest level until the zero level is achieved.

**Figure 3:**
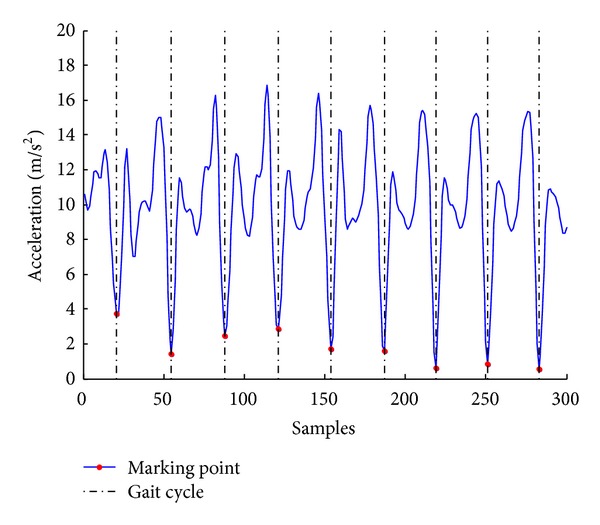
Gait cycle based segmentation on vertical dimension gait signal.

Because walking is a cyclic activity, we segment a sequence of gait signal after eliminating noise to separate patterns which consist of consecutive gait cycles. A gait cycle is defined as the time interval between two successive occurrences of one of the repetitive events when walking. We observed that whenever the human foot, which is on the same side as the device, touches the ground, the acceleration value in the vertical dimension signal changes obviously as illustrated as red points in Figure 3. We determined these points by calculating the autocorrelation coefficients *A*_*m*_ = ∑_*i*=1_^*N*−|*m*|^ *x*_*i*_ *x*_*i*+*m*_ on the vertical dimension signal and filtering vivid peaks based on mean and standard deviation. Then based on these points, we segment gait signals into separate patterns, in which each pattern consists of *n*_gc_  (*n*_gc_ = 4 in our experiment) consecutive gait cycles of all 3 dimensions. Finally, a feature vector is extracted from each pattern in both time and frequency domains.

### 3.2. Feature Vector Extraction

Denote *n*_gc_, *N* as the number of gait cycles (GC) and the number of acceleration values *x* in a pattern, respectively. In each pattern, gait features are extracted in both time and frequency domains as follows.

*(a) Time Domain Features.*

(i) Average maximum acceleration
  (2) $$\begin{array}{c} \text{av}{\text{g}}_{\max}=\text{mean}{\left(\max\left(\text{G}{\text{C}}_{i}\right)\right)}_{i=1}^{n_{\text{gc}}}. \end{array}$$
(ii) Average minimum acceleration
  (3) $$\begin{array}{c} \text{av}{\text{g}}_{\min}=\text{mean}{\left(\min\left(\text{G}{\text{C}}_{i}\right)\right)}_{i=1}^{n_{\text{gc}}}. \end{array}$$
(iii) Average absolute difference
  (4) $$\begin{array}{c} \text{av}{\text{g}}_{\text{abs}\_\text{dif}}=\overset{N-1}{\underset{i=0}{\sum }}\left|x_{i}-\;\bar{x}\right|. \end{array}$$
(iv) Root mean square
  (5) $$\begin{array}{c} \text{RMS}=\frac{1}{N}\overset{N-1}{\underset{i=0}{\sum }}x_{i}^{2}. \end{array}$$
(v) 10-bin histogram distribution
  (6) $$\begin{array}{c} \text{hd}={\langle n_{j}\rangle }_{0}^{9}\;\text{with}\;n_{j}=\frac{\sum_{i=0}x_{i}}{\text{size}\left(\text{bi}{\text{n}}_{j}\right)}\\ \frac{j\left(\max-\min\right)}{10}\leq x_{i}\in \text{bi}{\text{n}}_{j}<\frac{\left(j+1\right)\left(\max-\min\right)}{10}. \end{array}$$
(vi) Standard deviation
  (7) $$\begin{array}{c} \sigma =\sqrt{\left(\frac{1}{N-1}\right)\overset{N-1}{\underset{i=0}{\sum }}\left(x_{i}-\bar{x}\right)}. \end{array}$$
(vii) Waveform length
  (8) $$\begin{array}{c} \text{wl}=\overset{N-1}{\underset{i=1}{\sum }}\left|x_{i+1}-x_{i}\right|. \end{array}$$
(viii) Cadence period
  (9) $$\begin{array}{c} T_{\text{cad}}=\frac{\sum_{i}^{n}t\left(\text{G}{\text{C}}_{i}\right)}{n}, \end{array}$$
  where *t*() is the time length of a gait cycle.

*(b) Frequency Domain Features.*

(i) First 40 FFT coefficients
  (10) $$\begin{array}{c} \text{fft}=\langle X_{k}\rangle ,\;X_{k}=\overset{N-1}{\underset{i=0}{\sum }}x_{n}e^{-j2\pi ki/N}. \end{array}$$
(ii) First 40 DCT coefficients
  (11) $$\begin{array}{c} \text{dct}=\langle X_{k}\rangle ,\\ X_{k}=\frac{1}{2}x_{0}+\overset{N-1}{\underset{i=1}{\sum }}x_{i}\cos\left[\frac{\pi }{N}n\left(k+\frac{1}{2}\right)\right]. \end{array}$$

Note that each feature in time and frequency domains is extracted on 3 types of acceleration data of *Y*, *Z*, *M*-dimensions, where $a_{M}=\sqrt{a_{X}^{2}+a_{Y}^{2}+a_{Z}^{2}}$, except for the cadence period feature which is extracted based on the timestamp of acquired acceleration values. Totally, we obtain a real-valued feature vector of dimension of ((avg_max⁡_ + avg_min⁡_ + avg_abs_dif__ + RMS + hd + *σ* + wl + fft + dct) × 3 + *T*_cad_) = ((1 + 1 + 1 + 1 + 10 + 1 + 1 + 40+40) × 3 + 1) = 289 for each pattern.

### 3.3. Feature Vector Binarization

We adopt a quantization method which is previously used in [13] for face template binarization. Assume the number of users is denoted by *N*_*u*_. The number of feature vectors extracted from each user is *M*. Let ${\left(\vec{T}\right)}_{i,j}\;\left(i=1\cdots N_{u},j=1\cdots M\right)$ be the *j*th feature vector of the user *i*; the mean over intraclass variability ${\vec{\mu }}_{i}$ of the user *i* is calculated as

(12) $$\begin{array}{c} {\vec{\mu }}_{i}=\frac{1}{M}\overset{M}{\underset{j=1}{\sum }}{\vec{T}}_{j}. \end{array}$$

The mean over all feature vectors $\vec{\mu }$ in the enrollment phase is calculated by

(13) $$\begin{array}{c} \vec{\mu }=\frac{1}{N_{u}}\overset{N_{u}}{\underset{i=1}{\sum }}{\vec{\mu }}_{i}. \end{array}$$

The quantization method transforms *t*th component in ${\left(\vec{T}\right)}_{i,j}$ into {0,1} by comparing *t*th component of ${\vec{\mu }}_{i}$ with a specific threshold defined by corresponding* t*th component of *μ*. For each user *i*, the binary feature vector *ω*_*j*_ is determined by

(14) $$\begin{array}{c} {\vec{\omega }}_{i,j}={\langle \omega \rangle }_{t},\\ {\langle \omega \rangle }_{t}=\{\begin{array}{cc} 0 & \text{if}\;{\left(\overset{⟶}{\mu_{l}}\right)}_{t}\leq {\left(\vec{\mu }\right)}_{t}\\ 1 & \text{if}\;{\left(\overset{⟶}{\mu_{l}}\right)}_{t}>{\left(\vec{\mu }\right)}_{t}. \end{array} \end{array}$$

In the enrollment phase, we use enrollment feature vectors to approximately estimate the value of $\vec{\mu }$. This $\vec{\mu }$ is stored as the helper data and used as the specific threshold for binarizing real-valued feature vectors in the authentication phase.

### 3.4. Reliable Binary Feature Extraction

As the authors pointed out in [13], when using the quantization method to transform real-valued vectors into the binary forms based on statistical analysis as in the previous section, components in ${\vec{\omega }}_{i}$ are significantly instable when using $\overset{⟶}{\mu_{l}}$ and $\vec{\mu }$ to determine the output bit. For example, if the* t*th component of ${\left(\overset{⟶}{\mu_{l}}\right)}_{t}$ is close to ${\left(\vec{\mu }\right)}_{t}$, the error probability for the next verification will be higher. Therefore, it is necessary to extract only high robust and reliable bits among ${\vec{\omega }}_{i}$. First, the variance *σ* ^2^ of each* t*th component for each user *i* is calculated by

(15) $$\begin{array}{c} \sigma_{i,t}^{2}=\frac{1}{M-1}\overset{M}{\underset{j=1}{\sum }}{\left({\left({\vec{T}}_{i,j}\right)}_{t}-{\left(\overset{⟶}{\mu_{l}}\right)}_{t}\right)}^{2}. \end{array}$$

Assume that the variability of components is modeled as a Gaussian. Then, the standard error functions of* t*th bit of the user *i* are estimated as

(16) $$\begin{array}{c} {\text{rel\_val}}_{i}\left(t\right)=\frac{1}{2}\left(1+\mathrm{erf}\left(\frac{\left|{\left(\overset{⟶}{\mu_{l}}\right)}_{t}-{\left(\vec{\mu }\right)}_{t}\right|}{\sqrt{2\sigma_{i,t}^{2}}}\right)\right). \end{array}$$

Indices of rel_val_*i*_  (called rel_idx_*i*_) are also stored as the helper data to extract reliable bits in authentication phase.

### 3.5. Key Binding Scheme

We adopt the BCH code [20] as an error correcting code to overcome the natural variations between biometric measurements. The advantage of BCH code, compared with other codes, is that it can correct single errors which could occur randomly as in our extracted binary feature vectors. Moreover the decoding process of BCH code is designed to be simple. Therefore, it requires less computational capability and low-powered consumption so that our system is more lightweight to be possibly deployed on mobile devices. Let BCH_2_(*n*_*c*_, *k*, *t*) be a binary BCH code, where *n*_*c*_ is the code length of bits, *k* is the key length of bits, and *t* is the error correction capability. The binary cryptographic key *m* of length *k* is generated randomly corresponding to each user and then is encoded into the codeword *c* of length *n*_*c*_ using a BCH_2_(*n*_*c*_, *k*, *t*) encoding scheme [20]. After that, we conceal this *c* by binding it with the extracted binary feature vector *ω* yielding a secured *δ* and then discard *ω*. Since *ω*, *c* are two binary strings, an exclusive*-*OR operator is adopted to bind these two strings together.

In summary, we represent all of the necessary steps in both enrollment and authentication phases in our system as follows.

*Enrollment Phase.*

- Select a BCH_2_(*n*_*c*_, *k*, *t*) by predefining parameters including the length *n*_*c*_ of the codeword and the length *k* of the secret key.
- For each user *i*, real-valued feature vectors *T*_*i*_ ∈ *R* ^*n*_*r*_^  are extracted.
- Determine a mean over all feature vectors $\vec{\mu }$ and extract a binary vector *ω*_*i*_ ∈ {0,1}^*n*_*r*_^  by using the quantization scheme. Then, discard *T*_*i*_.
- Determine the reliable bit indices rel_idx_*i*_ and reduce the length of *ω*_*i*_ to *n*_*c*_  by only selecting first *n*_*c*_ bits among *n*_*r*_ based on rel_idx_*i*_.
- Store $\vec{\mu },{\text{rel\_idx}}_{i}\;$as helper data for further use to construct new feature vectors in the authentication phase.
- Randomly generate a binary secret key *m*_*i*_ with the length of *k*.
- Calculate the hash value of *m*_*i*_ by using a cryptographic hash function *h* (e.g., SHA) and store *h*(*m*_*i*_).
- Encode *m*_*i*_ using a BCH_2_(*n*_*c*_, *k*, *t*) encoding scheme to obtain a codeword *c*_*i*_. Then, discard *m*_*i*_.
- Bind *c*_*i*_ with *ω*_*i*_ using exclusive-OR operator yielding *δ*_*i*_. Then, discard *ω*_*i*_ and store *δ*_*i*_.

*Authentication Phase*.

- For each user *i*, feature vectors *T*_*i*_′ ∈ *R* ^*n*_*r*_^ are extracted from a new biometric sample.
- Extract binary feature vectors *ω*_*i*_′  with length of *n*_*c*_ with the help of $\vec{\mu }\;$and *rel*⁡_idx_*i*_. Then, discard *T*_*i*_′.
- Bind *ω*_*i*_′  with the stored *δ*_*i*_ using exclusive-OR operator to obtain a corrupted codeword *c*_*i*_′.
- Decode *c*_*i*_′ using a BCH decoding scheme to obtain a key *m*_*i*_′ from  *c*_*i*_′.
- Calculate hash value *h*(*m*_*i*_′) using the equivalent cryptographic hash function (e.g., SHA) as in the enrollment phase and then discard *m*_*i*_′.
- Match *h*(*m*_*i*_) with *h*(*m*_*i*_′); if *h*(*m*_*i*_) = *h*(*m*_*i*_′), the user *i* is authenticated. Otherwise, he will be rejected.

## 4. Experiments

### 4.1. Dataset Description

We evaluate our system on the data collected from a built-in accelerometer in Google Nexus One mobile phone. The sampling rate of the sensor is approximately 27 Hz by setting to SENSOR_DELAY_FASTED mode on Android SDK. A total of 34 volunteers including 24 males and 10 females with the average age from 24 to 28 participated in our dataset construction. Each volunteer will perform around 18 laps. To make the dataset more realistic, we collect gait signals regardless of footgear and clothes. Volunteers are asked to walk as naturally as possible and change their footgear (e.g., sandal, shoe, or slipper) as well as clothes (e.g., short to long trouser, etc.) whenever they start a new lap. We only have a constraint that when volunteers perform walking, the mobile put in the pocket will not change its position and orientation. To ensure that, we request volunteers to wear trousers having a narrow pocket. Totally, we accumulated the gait signals of 34 volunteers, each having at least 16 real-valued feature vectors which could be extracted using the method in Section 3.2. In our experiment, each volunteer will have an equal number of the extracted feature vectors so that we randomly select 16 vectors for users having more than 16.

### 4.2. Results

**Figure 4:**
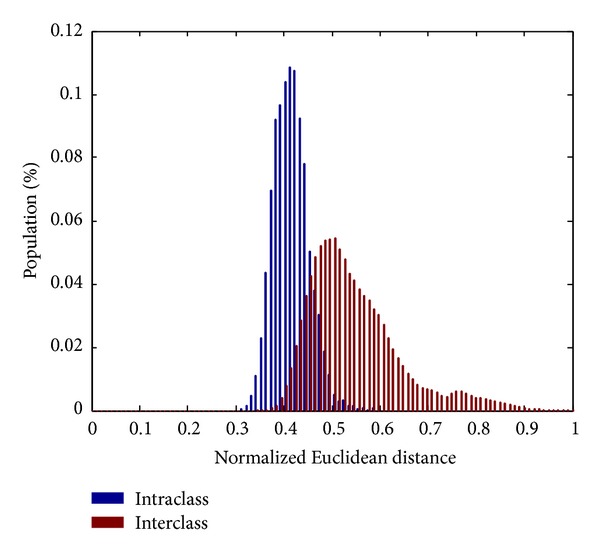
The Euclidean distance of extracted intra- and interclass feature vectors.

**Figure 5:**
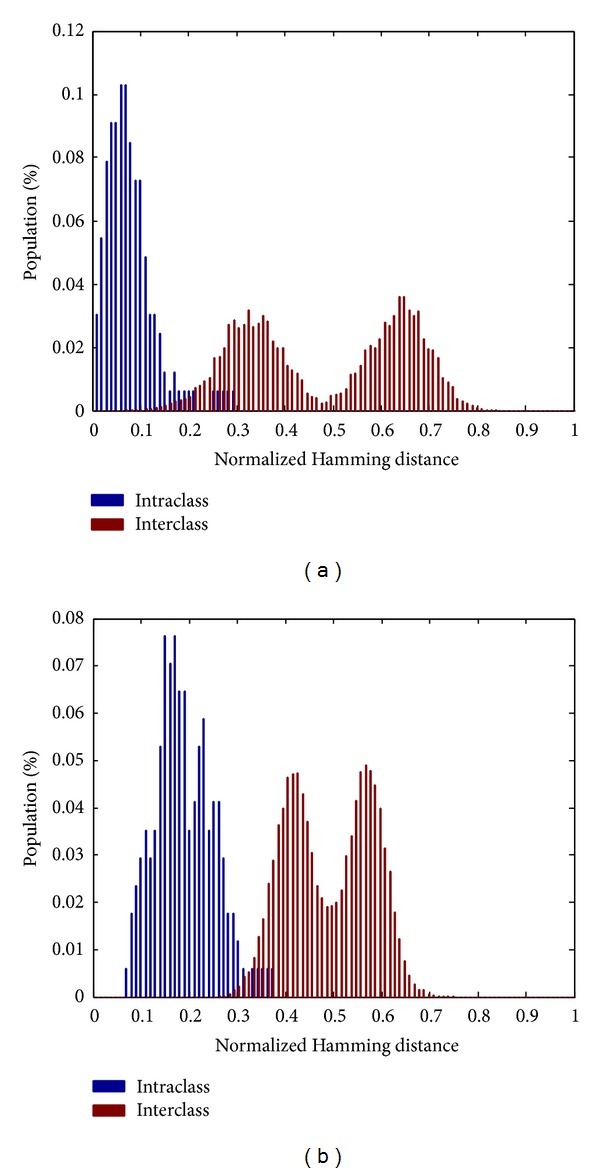
The Hamming distance of intra- and interclass binary vectors of lengths of 127 (a) and 255 (b).

Figure 4 represents the Euclidean distance distribution of extracted real-valued feature vectors. Note that the operation of our BCS is likely to be similar to a threshold-based classification, in which the threshold is likely to be low according to an appropriate distance metric. We can see that the mixing area between intraclass and interclass real-valued feature vectors is large. Thus, applying threshold based classification on these vectors would lead to the high error rate in terms of FAR and FRR. Fortunately, when such vectors are binarized by using the proposed method in Section 3.3, the discrimination of binary feature vectors between users is likely to be higher and the Hamming distance of intraclass feature vectors is getting lower.  Figure 5 illustrates the Hamming distance of binary feature vectors of lengths of 127 and 255, respectively. These values of length are selected to be appropriate with the design of the BCH code which allows the length of codeword to be equal to 2^*M*^ − 1, *M* ∈ *N*, *M* > 3 and the maximum dimension *d*_max⁡_ of feature vector which could be extracted in this study (*d*_max⁡_ = 289). As already stated, the length of binary feature vector must be equal to the length of BCH codeword for possible binding using an Exclusive*-*OR operator. Hence, the reliable bit extraction process in Section 3.4 will only select a number of reliable components identical to the codeword length. Looking into Figure 5, we can see that the Hamming distance of intraclass feature vectors of length of 127 is lower than in case of length of 255. We found that this is due to the fact that the actual number of bits being highly reliable according to (16) is just approximately half of the original feature vector dimension. Hence, to obtain a binary feature vector of length of 255, even low reliable bits are also selected.

**Figure 6:**
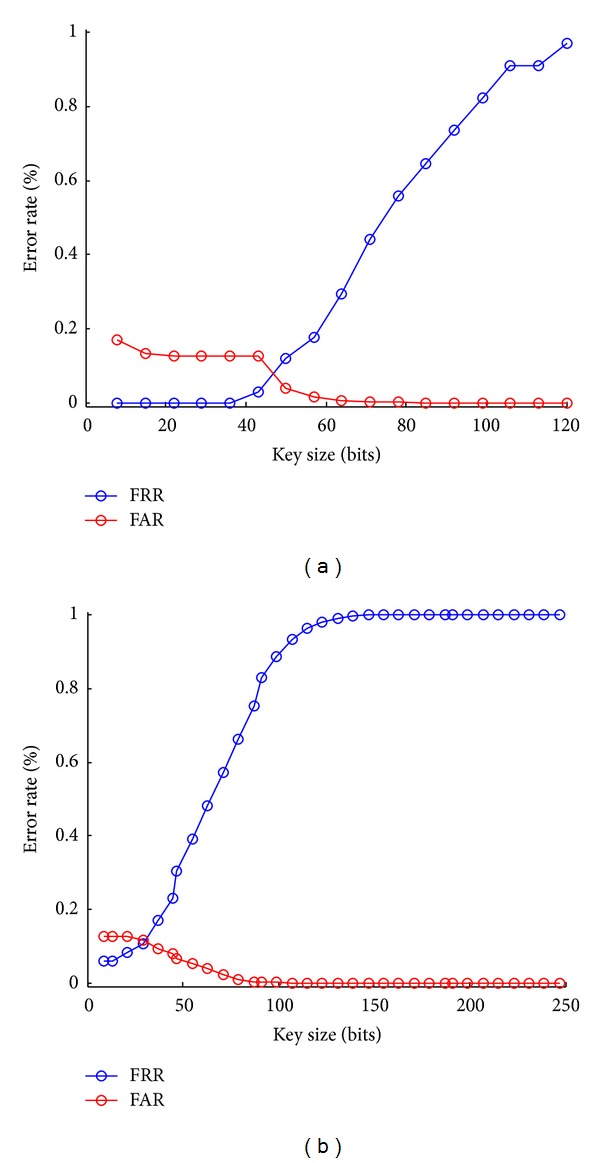
The error rates of FAR and FRR of the key binding result in terms of codeword lengths of 127 (a) and 255 (b).

Figure 6 illustrates the error rates of our proposed gait based BCS using fuzzy commitment scheme corresponding to two codeword lengths of 127 and 255, respectively. In both cases, when the key length increases which is equivalent to the number of errors allowed in the codeword decreases, the FAR is getting reduced to 0 and the FRR exponentially increases. The best error rates of our proposed system are (1) in the case of codeword length = 127; the achievements of FAR and FRR are approximately 3.921% and 11.76%, respectively, in terms of key length = 50 bits. (2) In the case of codeword length = 255, we achieve the FAR *≈* 1.4% and the FRR *≈* 32.53% in terms of the key length = 55 bits. These keys are rather sufficiently long to be secured by a cryptographic hash algorithm. The FRR of codeword length = 255 is significantly higher than in case of codeword length = 127 because, as already stated, selecting many low reliable bits makes the binary feature vectors of length = 255 more dissimilar. However, the achieved FAR is slightly better (1.4% compared with 3.921%). In both cases, we can see that the FRRs are rather high which could decrease the friendliness of the system. However, user's gait could be captured continuously and implicitly by an accelerometer which does not make the user annoyed as other biometric modalities (e.g., iris, fingerprint, face, and signature). Therefore, this issue is not so considerable.

Table 1 shows the performance of our proposed system compared to some other state-of-the-art BCSs using different behavioral modalities such as voice and signature. Note that all these works use different approaches and the dataset used is totally different so the comparison is just relative. Therefore, through this study, we would merely like to illustrate that human gait captured from inertial sensors could be utilized to construct an effective BCS as other behavioral modalities. Moreover due to the fact that we adopt a quantization scheme similar to [13], we also compare our system with this face based BCS. The authors achieved the key length of 58 bits, the FAR of approximately 0%, and the FRR of approximately 3.5% and 35% corresponding to two different datasets of CALTECH and FERET, respectively. We can see that face is a physiological biometric which is more robust than human gait, which is a behavioral modality. Hence, the performance of their system in terms of key length, FAR, and FRR is slightly better.

## 5. Conclusion

In this paper, we introduce an approach of gait based biometric cryptosystem using fuzzy commitment scheme. The results show a good potential to construct an effective gait based BCS especially on mobile devices. The drawbacks of our work are that the error rates in terms of FAR and FRR are still rather high. We expect to achieve the FAR of 0% to make the system more secured. Hence, our further work will focus on reducing the error rates of FAR and FRR by constructing higher discriminant feature vectors using global feature transformations as well as finding an optimal quantization scheme for binarization. Moreover, the system security should be analyzed in depth to ensure that a gait based biometric cryptosystem could fulfill the security requirement in order to be deployed in reality. Finally, validating the proposed system on a larger public dataset is also our main further work.

## Figures and Tables

**Table 1:** Relative comparison of our proposed system and state-of-the-art BCSs using different schemes of fuzzy commitment scheme (FCS) and fuzzy extractor (FE).

Study	Modality	Scheme	Key size (bits)	FAR (%)	FRR (%)
[13]	Face (CALTECH) (FERET)	FCS	58 58	*≈*0 *≈*0	3.5 35
[15]	Signature	FCS	29	6.95	6.95
[16]	Voice	FE	30–51	<10	<10
This study	Gait	FCS	55 50	3.92 1.4	11.76 32.53

## References

[1] Augusto JC, Callaghan V, Cook D, Kameas A, Satoh I (2013). Intelligent Environments: a manifesto. *Human-Centric Computing and Information Sciences*.

[2] Tsai CL, Chen CJ, Zhuang DJ (2012). Trusted M-banking Verification Scheme based on a combination of OTP and Biometrics. *Journal of Convergence*.

[3] Ng JKY (2012). Ubiquitous healthcare: healthcare systems and applications enabled by mobile and wireless technologies. *Journal of Convergence*.

[4] Ahn J, Han R (2012). An indoor augmented-reality evacuation system for the Smartphone using personalized Pedometry. *Human-Centric Computing and Information Sciences*.

[5] Teraoka T (2012). Organization and exploration of heterogeneous personal data collected in daily life. *Human-Centric Computing and Information Sciences*.

[6] Birgale L, Kokare M (2012). Iris recognition using ridgelets. *Journal of Information Processing Systems*.

[7] Bharkad SD, Kokare M (2012). Hartley transform based fingerprint matching. *Journal of Information Processing Systems*.

[8] Satone MP, Kharate GK (2012). Face recognition based on PCA on wavelet subband of Average-Half-Face. *Journal of Information Processing Systems*.

[9] Hoang T, Nguyen T, Luong C, Do S, Choi D (2013). Adaptive cross-device gait recognition using a mobile accelerometer. *Journal of Information Processing Systems*.

[10] Juels A, Wattenberg M Fuzzy commitment scheme.

[11] Rathgeb C, Uhl A (2011). A survey on biometric cryptosystems and cancelable biometrics. *EURASIP Journal on Information Security*.

[12] Álvarez Mariño R, Álvarez FH, Encinas LH (2012). A crypto-biometric scheme based on iris-templates with fuzzy extractors. *Information Sciences*.

[13] van der Veen M, Kevenaar T, Schrijen G-J, Akkermans TH, Zuo F Face biometrics with renewable templates.

[14] Li P, Yang X, Qiao H, Cao K, Liu E, Tian J (2012). An effective biometric cryptosystem combining fingerprints with error correction codes. *Expert Systems with Applications*.

[15] Maiorana E (2010). Biometric cryptosystem using function based on-line signature recognition. *Expert Systems with Applications*.

[16] Carrara B, Adams C You are the key: generating cryptographic keys from voice biometrics.

[17] Hao F, Anderson R, Daugman J (2006). Combining crypto with biometrics effectively. *IEEE Transactions on Computers*.

[18] Dodis Y, Reyzin L, Smith A (2004). Fuzzy extractors: how to generate strong keys from biometrics and other noisy data. *Advances in Cryptology-Eurocrypt 2004*.

[19] Li Q, Sutcu Y, Memon N (2006). Secure sketch for biometric templates. *Advances in Cryptology-ASIACRYPT 2006*.

[20] Morelos-Zaragoza RH (2002). *The Art of Error Correcting Coding*.

